# Through Different Lenses: A Retrospective Analysis of the Agreement Between Laparoscopic and Histopathological Evaluations of the Appendix

**DOI:** 10.7759/cureus.65785

**Published:** 2024-07-30

**Authors:** Christine-Bianca Hanganu, Sanad Isswiasi, Abiodun Adigun, Kyrllos Farag, Vladimir Nichita, Muhammadhasan Anwaar, Ahmed Esawi, Rishi Sen, Akshay Bavikatte, Elisabeth Drye

**Affiliations:** 1 General Surgery, Cambridge University Hospitals NHS Foundation Trust, Cambridge, GBR; 2 General Surgery, West Suffolk Hospital, Bury St Edmunds, GBR; 3 Cardiothoracic Surgery, Manchester University NHS Foundation Trust, Manchester, GBR; 4 General Surgery and Oncoplastic Breast Surgery, West Suffolk Hospital, Bury St Edmunds, GBR; 5 General Surgery, Peterborough City Hospital, Peterborough, GBR; 6 Colorectal Surgery, West Suffolk Hospital, Bury St Edmunds, GBR

**Keywords:** appendicectomy, post-operative histopathology, laparoscopic diagnosis, vermiform appendix, laparoscopic assessment, histopathology report, appendicitis

## Abstract

Background: Appendicectomy is the most frequently performed emergency general surgical procedure. Previous research has highlighted discrepancies between initial intraoperative laparoscopic diagnoses and subsequent histopathology reports following appendicectomy. In the United Kingdom (UK), routine histopathological examination is the established practice, ensuring precise diagnosis of appendiceal specimens. This retrospective analysis aims to compare intraoperative laparoscopic assessments of the appendix with corresponding histopathology findings.

Methodology: We conducted a retrospective analysis of 418 consecutive emergency laparoscopic appendectomies at Peterborough City Hospital in the UK between April 2018 and June 2019 for suspected appendicitis. Intraoperative findings were compared with histopathological examination outcomes using kappa statistics.

Results: Of the 418 appendectomies analysed, we found a substantial agreement between surgeons and pathologists (kappa = 0.71, P < 0.001) in detecting overall abnormalities of appendices. This agreement was also high for detecting inflamed appendices (kappa = 0.72, P < 0.001). However, of the six neoplastic lesions confirmed in the pathologists' final report, only two were suspected during laparoscopy.

Conclusions: While laparoscopic assessments of the appendix demonstrate a statistically significant and improved agreement with histopathological findings in detecting abnormal and inflamed appendices, the ability of surgeons to identify neoplasia appears suboptimal based on our small sample of neoplasia cases. The data strongly support the continued practice of routine histopathological examination following appendicectomy due to its crucial role in avoiding missed diagnoses and ensuring better patient outcomes.

## Introduction

Acute appendicitis continues to represent the predominant general surgical emergency in the United Kingdom (UK), accounting for approximately 50,000 cases undergoing appendicectomies annually [1]. Within our institution, the standard protocol involves the routine histopathological analysis of appendiceal specimens.

Numerous studies have documented significant disparities between initial intraoperative assessments and subsequent histopathological findings. These discrepancies highlight the potential risk that surgeons' evaluations may inadvertently miss clinically significant pathologies such as inflammatory disorders, parasitic infestations, and neoplasms, all of which can have a significant impact on patient well-being [2-14].

However, some studies argue that routine histopathological examination is unnecessary unless there are obvious macroscopic abnormalities observed during the operation [15-18]. These studies suggest that this practice is justified due to the rarity of incidental findings, the costs associated with specimen processing, and the infrequent need to change management based on the histology report following an appendicectomy.

The aim of this study was to compare the current intra-operative laparoscopic assessment of the appendix during laparoscopic surgery with the corresponding histopathology findings in our centre.

## Materials and methods

We conducted a retrospective review at Peterborough City Hospital, North West Anglia NHS Foundation Trust, UK, analysing 418 consecutive emergency laparoscopic appendicectomies for suspected appendicitis from April 2018 to June 2019. Patients were identified using the hospital’s coding system and cross-referenced with the electronic theatre operating system. Patients who underwent open or laparoscopic-converted to-open procedures were excluded.

Data on patient demographics, including age and gender, length of stay, use of antibiotics, and histopathological request forms and reports of the appendicectomy specimens were extracted from the Sunquest ICE database (Sunquest Information Systems Inc., Tucson, AZ). Outcomes and follow-up communication letters were also reviewed using our online system.

Statistical analyses were performed using Statistical Product and Service Solutions (SPSS, version 20; IBM Corp., Armonk, NY) software. The agreement between surgeons' assessments and pathologists' evaluations was measured using the Cohen kappa statistics [19], with values interpreted as follows: < 0.20 indicated slight agreement, 0.21-0.40 fair agreement, 0.41-0.60 moderate agreement, 0.61-0.80 substantial agreement, and 0.81-1.00 almost perfect agreement. Statistical significance was calculated by SPSS using the Z-test, with a P-value < 0.05 considered significant. This study was formally registered and accepted by the Quality Governance and Compliance Department at North West Anglia NHS Foundation Trust, UK.

## Results

We included a total of 418 patients in the study, with a median age of 25 years and an equal male-to-female ratio. Among them, 295 patients (70.6%) were older than 17 years. Computed tomography (CT) scans were performed on 79 patients (18.9%). The median hospital stay was one day, and patients received antibiotics for a median duration of three days. Laparoscopic examinations described 328 specimens (78.5%) as abnormal, with 323 appendices (77.3%) identified as inflamed, including 80 (19.1%) with perforation. Table 1 summarises patient characteristics and findings.

**Table 1:** Summary of Patient Characteristics and Findings Data represented as n (total=418), (%), and median with range (range) (where mentioned).

Characteristics		Value
Median age in years, median (range)		25 (4-88)
Female vs. male, n (%):n (%)		210 (50.3):208 (49.7)
>17-year-old, n (%)		295 (70.6)
CT scan performed, n (%)		79 (18.9%)
Laparoscopic findings	Normal appendix, n (%)	90 (21.5)
	Abnormal appendix, n (%)	328 (78.5)
	Inflamed appendix, n (%)	323 (77.3)
	Perforated appendix, n (%)	80 (19)
Patients with normal histopathology, n (%)		107 (25.6)
Female vs. Male with a normal appendix on histopathology, n (%):n (%)		74 (17.7):33 (7.9)
Median length of stay in days, median (range)		1 (1-5)
Median number of days on antibiotics, median (range)		3 (0-16)

**Figure 1:**
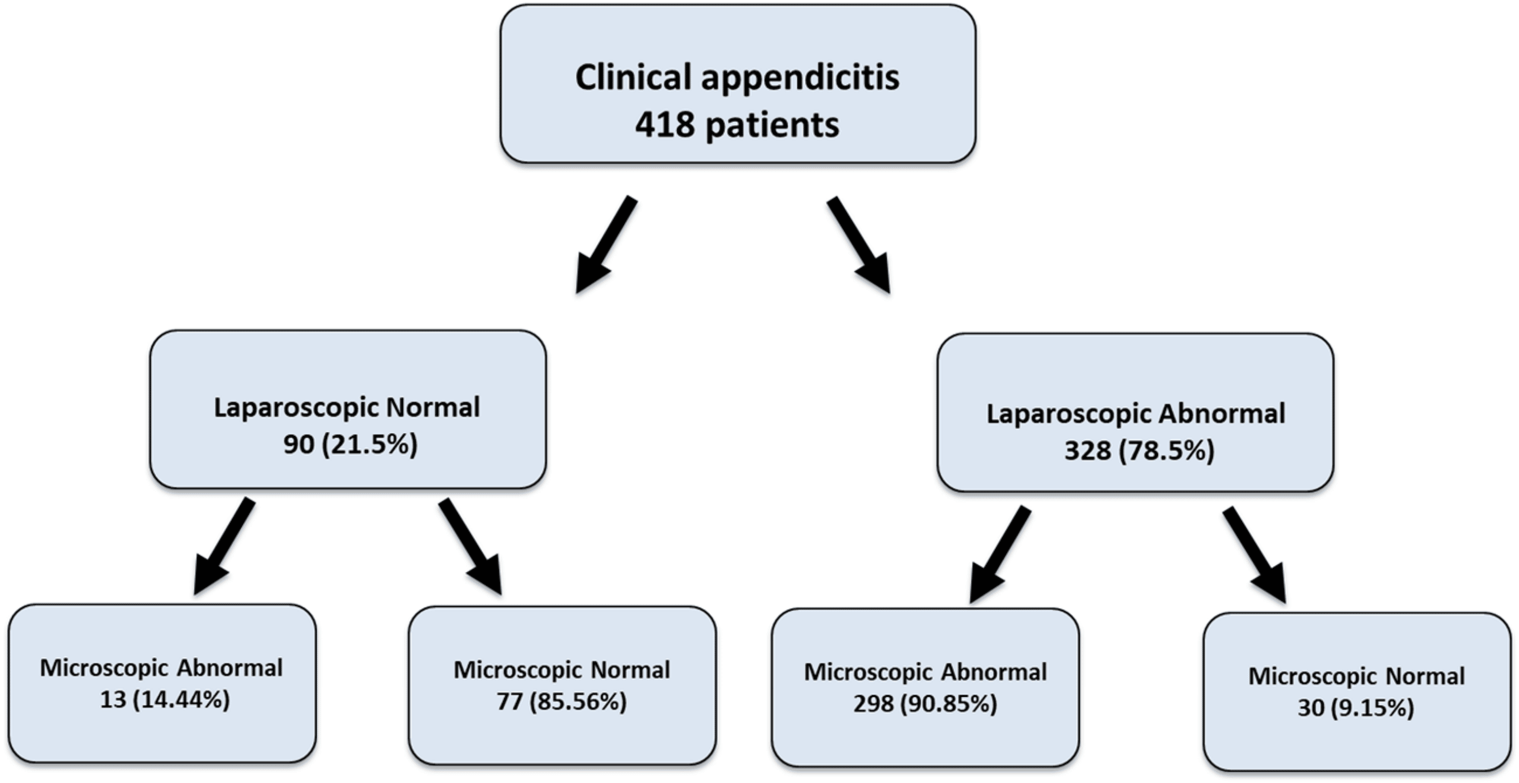
Flow Chart of the Laparoscopic and the Microscopic Examination

Out of 90 patients (21.5%) identified as having a normal appendix laparoscopically, 77 (85.56%) were confirmed to be normal microscopically, while 13 (14.44%) were found to be abnormal findings on the histology reports. Among the 328 patients (78.5%) with laparoscopically abnormal appendices, 298 (90.85%) were confirmed to be abnormal microscopically, whereas 30 (9.15%) were found to be normal microscopically. The overall level of agreement between laparoscopic and histopathological findings in identifying abnormal appendices was substantial (kappa = 0.714, P < 0.001). Figure 1 illustrates the flowchart for the laparoscopic versus histopathological diagnosis of appendicitis.

The surgeons suspected cancer in the pathology request forms for five laparoscopies. Upon histological microscopic analysis, two of these were histologically reported as mucinous lesions, while the remaining were inflamed. Further microscopic assessments revealed several specific pathologies, as shown in Table 2. These included three mucinous lesion, one neuroendocrine tumour, one secondary deposit from pancreatic adenocarcinoma, and one appendiceal polyp. Only the three mucinous lesions were suspected by the pathologists’ macroscopic analysis. Importantly, two of our locally resected specimens necessitated second opinions from tertiary centres, and all patients, except for the one with an appendiceal polyp, required either further treatment or surveillance, as shown in Table 2.

**Table 2:** Summary of Other Detected Pathologies and Corresponding Further Management * Two of those three mucinous lesions were suspected by surgeons.

Final diagnoses by pathologists (microscopic examination)	Number of pathologies in the final pathology report	Further management after appendicectomy
Mucinous lesions	3*	Two offered surveillance and one offered further resection
Neuroendocrine tumour	1	Further resection
Metastatic pancreatic adenocarcinoma	1	Palliative chemotherapy
Appendiceal polyp	1	No further treatment
Enterobius vermicularis	4	Antihelminthic treatment

A subgroup analysis was conducted to assess the agreement between laparoscopic and histopathological evaluations regarding the presence of inflammation. This analysis excluded specimens that were laparoscopically suspected or/and histologically confirmed to have pathologies other than inflammation (n =13). The agreement between laparoscopic and histopathological findings in detecting an inflamed appendix was substantial (kappa = 0.72, P < 0.001).

## Discussion

Our study demonstrates a substantial agreement between laparoscopic assessments and histopathological findings in evaluating appendiceal specimens for all appendiceal abnormalities (kappa = 0.71, P < 0.001) and appendicitis (kappa = 0.72, P < 0.001).

In comparison to a prospective multi-centre cohort study from the UK by Strong et al. [6], which involved more than 3,000 appendicectomies and reported a moderate overall agreement (kappa = 0.571) between the assessments made by surgeons and pathologists, our study observed a higher agreement (kappa = 0.71). The improved agreement in our study could be attributed to advancements in technology, such as enhanced laparoscopes and monitors, as well as increased training and experience among surgeons.

We reported that surgeons suspected two out of the six neoplastic lesions. Macroscopically, pathologists suspected three of those six lesions, including the two lesions suspected by surgeons. All three of these lesions were mucinous. It is notable that only one case, involving an appendiceal polyp, did not necessitate additional follow-up, resection, or chemotherapy. This underscores the ongoing clinical importance of routine microscopic examinations of appendiceal specimens to ensure accurate diagnoses and improve patient outcomes. Alemayehu et al. highlighted this limitation of macroscopic assessment of appendices, reporting that less than 5% of unusual pathology was anticipated [18]. Some authors have noted interobserver disagreement among pathologists and speculated that surgeons’ initial opinions on specimens may influence subsequent pathology reports, particularly for appendiceal specimens [20]. Notably, two of our locally resected specimens required second opinions from tertiary centres.

Conversely, some studies argue against routine histopathological examination unless gross macroscopic pathology is observed during the operation [15-18]. These studies suggest that this practice is justified, due to the rarity of incidental findings, the costs associated with specimen processing, and the infrequent need to change management based on the histology report following an appendicectomy. A recent audit from the UK reported that more than 100 appendiceal specimens were not reviewed by surgeons, with no significant health or medico-legal implications after the operation [21]. A multicentric prospective analysis conducted in the Netherlands [16] involving 7,339 patients, included thorough inspection and palpation of the appendix and mesoappendix by surgeons. They found that 67.7% of the specimens would have been deemed unnecessary for histopathological examination, as only 22 out of 4,966 patients had appendiceal neoplasms. It is worth noting that in this study, a consultant surgeon was present in >50% of the procedures, and the specimens were thoroughly inspected and palpated after resection. These practices, however, are not widely popular in the UK.

Nevertheless, failing to acknowledge significant test results can lead to serious health implications and potential allegations of medical negligence [22-24]. A UK-based study by Mosedale et al. reported that 2% of medicolegal claims following appendicectomy were related to inadequate follow-up, resulting in a median payout of £8,205 per case by the NHS between 2002 and 2011 [24]. The study also found that a lack of appropriate response to histological reports indicating malignancy led to a 100% success rate in litigation.

Our findings highlight the improved agreement between laparoscopic and histopathological reports in detecting overall abnormality and inflammation of the appendix. Nevertheless, the critical role of routine histopathological examinations in detecting neoplasia remains clinically essential to ensure accurate diagnosis, reliable follow-up, and safe patient care. However, our analysis is constrained by its retrospective and non-randomised design, as well as being based on the experience of a single hospital. While the overall sample size is considerable, it remains insufficient to adequately examine the agreement on neoplastic lesions between surgeons and pathologists.

## Conclusions

While laparoscopic assessments of the appendix demonstrate a statistically significant and improved agreement with histopathological findings in detecting abnormal and inflamed appendices, the ability of surgeons to identify neoplasia appears suboptimal based on our small sample of neoplasia cases. The data strongly support the continued practice of routine histopathological examination following appendicectomy due to its crucial role in avoiding missed diagnoses and ensuring better patient outcomes.
